# Beyond economic outcomes: Exploring the social contributions of skills training for persons with disabilities in South Africa

**DOI:** 10.4102/ajod.v15i0.2072

**Published:** 2026-08-29

**Authors:** Johannes A. Olivier-Booysen, Maximus M. Sefotho

**Affiliations:** 1 Faculty of Education, University of Johannesburg, Johannesburg, South Africa

**Keywords:** disability and employment, empowerment, persons with disabilities, qualitative research, quality of life, skills development, social inclusion, South Africa

## Abstract

**Background:**

Persons with disabilities experience significant economic exclusion in South Africa. While vocational training has often been viewed through a narrow economic lens, its broader social contributions remained under-researched.

**Objectives:**

This study examined the social contributions of skills training for persons with disabilities, with specific attention to empowerment, inclusion and well-being.

**Method:**

A qualitative multiple-case study design was employed. Data were generated through semi-structured interviews, photovoice, document analysis and field notes with eight participants across three South African provinces.

**Results:**

Training contributed to psychological empowerment, increased independence and improved quality of life. Participants reported enhanced self-worth and a stronger sense of purpose. However, persistent structural and attitudinal barriers continued to constrain full social and economic participation.

**Conclusion:**

Skills training functioned as a catalyst for personal transformation and social belonging. Nevertheless, translating acquired skills into sustained participation required addressing broader environmental constraints and gaps in policy implementation.

**Contribution:**

This study extended disability scholarship by foregrounding the holistic, non-economic value of training interventions in fostering dignity, agency and social inclusion among persons with disabilities.

## Introduction

Entrepreneurship education may strengthen entrepreneurial self-efficacy, intention and readiness for economic participation (Bux & van Vuuren 2019; Chimucheka 2014; Usman & Widyanti 2020). In South Africa, persons with disabilities (PWDs) continue to encounter obstacles when transitioning from education and training into professional and employment environments (Ndlovu & Walton 2016). Globally, and specifically within South Africa, PWDs continue to experience disproportionately high levels of unemployment, economic exclusion and social marginalisation (Statistics South Africa 2024; World Health Organization 2023). Limited access to inclusive employment and training has contributed to persistent inequalities in income, well-being and social participation. In response, skills-based and vocational training interventions are increasingly promoted as pathways to economic engagement and social inclusion, particularly in sectors where applied competencies may reduce structural barriers to entry (Mitra, Posarac & Vick 2013). The homebuilding industry presents a significant opportunity for economic participation, yet PWDs in this sector face intensified exclusion because of physical demands, regulatory complexity and persistent stigma regarding their competence (Maziriri, Madinga & Lose 2017; Morwane, Dada & Bornman 2021). Despite growing interest in disability-inclusive workforce development, existing research in South Africa has largely focused on immediate economic outcomes and structural barriers to labour market entry (Engelbrecht, Morris & Ned 2026; Shaw et al. 2022). There remains a significant knowledge gap regarding the holistic, non-economic social contributions of training interventions, such as psychological empowerment, social belonging and enhanced quality of life. This study addresses this gap by investigating how training catalyses personal and social transformation within the homebuilding industry. By examining these post-training social contributions, the research seeks to extend disability scholarship beyond narrow economic indicators towards more comprehensive understandings of inclusion and agency. The home building industry provides a relevant context for this study because of its historically exclusionary nature, where physically demanding work and entrenched assumptions about ability often limit participation of PWDs. At the same time, the sector has increasingly been targeted for skills development and transformation initiatives, creating opportunities to explore how training contributes to inclusion within a structurally constrained environment (Bailey et al. 2022). This study is grounded in the social model of disability, which understands disability as arising from social, environmental and attitudinal structures rather than individual impairment (Barnes 2019; Bunbury 2019). To examine the intersection of career development and disability, the study utilises the Career Construction for Hephapreneurship (CCH) framework. Career-development interventions may also strengthen graduates’ employability capabilities and capacity to navigate restrictive labour markets (Otu & Sefotho 2024). Developed by Sefotho (2013), CCH integrates career guidance, disability studies and public policy to emphasise ‘hephapreneurship’ as the capacity of PWDs to ‘take charge’ and become self-sufficient. This framework aligns with empowerment-oriented and positive psychology frameworks that seek to affirm the competencies of PWDs in social and economic contexts (Leshota & Sefotho 2020; Sefotho 2013).

The primary aim of this study is to explore the social contributions of skills training for PWDs in the South African homebuilding industry. To achieve this, the study addresses the following objectives:

- To explore how skills training contributes to the empowerment, independence and well-being of PWDs.
- To examine how skills training influences social inclusion and economic participation among PWDs.

## Research methods and design

### Study design

The study was guided by a constructivist–interpretivist paradigm, which assumes that meaning is socially constructed through participants’ lived experiences and interpretations of their environments. Within this paradigm, knowledge is understood as emerging from participants’ subjective experiences and the meanings they assign to social realities. A qualitative multiple-case study design was employed to gain an in-depth understanding of the social contributions of training for PWDs in the homebuilding industry. This design enabled exploration of participants’ post-training experiences across multiple real-life contexts, providing rich, contextually grounded insight into how training outcomes were interpreted, enacted and sustained in everyday settings. The use of multiple cases further allowed the study to capture similarities and variations in experiences across participants, thereby strengthening the depth and credibility of the findings.

### Study setting and population

The study was conducted across three South African provinces (Gauteng [GP], the Western Cape [WC], and North West [NW]), selected based on the availability of PWDs who had previously participated in homebuilding sector training. The training included short skills and entrepreneurship-oriented programmes focused on construction competencies and small business development. The study population comprised adult PWDs who had completed the training and were able to meaningfully reflect on their post-training experiences during data collection. The broader population of PWDs who have participated in homebuilding-related training across South Africa is not formally consolidated within a single accessible database, as such training is offered across multiple programmes and contexts. As a result, the study focused on a purposively selected group of participants who met the inclusion criteria and were accessible for in-depth qualitative engagement. Recruitment was conducted through purposive and snowball sampling to identify individuals with relevant training experience. Data saturation was reached after eight interviews, as no new themes emerged, which is consistent with qualitative research approaches that prioritise depth and richness of data over sample size. The inclusion of participants from multiple provinces enabled the exploration of diverse lived experiences shaped by different social, economic and infrastructural contexts within South Africa.

### Inclusion and exclusion criteria

Participants were included if they identified as PWDs, were adults capable of providing informed consent and had previously completed training relevant to the homebuilding industry. Participants were required to engage meaningfully in an interview and reflect on their post-training experiences. Individuals who had not participated in homebuilding sector training or who were unable to participate in an interview meaningfully were excluded from the study.

### Sample size

Sampling refers to selecting a subset of participants to explore key characteristics and experiences relevant to the research focus (Karunarathna et al. 2024). In this qualitative multiple-case study, data collection continued until thematic saturation was reached, indicating that no new themes or insights were emerging from the interviews. Saturation was achieved after eight interviews, which provided sufficient depth and richness to examine the social contributions of training. This sample size aligns with qualitative research approaches that prioritise analytic depth and interpretive richness over numerical representation (Ahmed 2025; Creswell & Creswell 2017).

### Sampling technique

A purposive sampling technique was employed to identify participants who met the study’s specific inclusion criteria. This approach was appropriate given the specialised characteristics of the target group and the exploratory nature of the research. Where necessary, participants were invited to recommend others with similar training experiences, enabling limited snowball sampling to support recruitment (Creswell & Creswell 2017; Cyr & Goodman 2024). The sample comprised eight adult PWDs (P1–P8) from three South African provinces. Participants varied in age, type of disability and training experience within the home building sector. Disabilities included physical, sensory and other functional impairments. Participants had completed training in areas such as construction-related skills and small business development and were at different stages of post-training engagement, including informal employment, self-employment or continued job-seeking. This diversity enabled the study to capture a range of perspectives on the social contributions of training.

### Data collection

To enhance the credibility and depth of the findings, data were collected from multiple sources. Data were collected through semi-structured interviews, photovoice submissions, document analysis and field notes to provide a comprehensive understanding of participants’ lived experiences. The use of multiple forms of evidence strengthened accuracy and supported a more comprehensive understanding of participants’ lived experiences. Triangulation across data sources supported data saturation and enhanced the trustworthiness of the findings.

#### Individual interviews

Semi-structured individual interviews served as the primary data source. A semi-structured interview guide was developed to explore participants’ experiences of empowerment, social inclusion, well-being and post-training economic participation. The development of the interview guide was informed by the study’s theoretical framework, focusing on constructs such as empowerment, agency and social inclusion. This approach allowed flexibility, enabling the researcher to probe, clarify and adapt questions in response to participants’ experiences (Kvale & Brinkmann 2015; Patton 2023). Open-ended questions encouraged participants to describe their experiences freely, while the researcher maintained a conversational and responsive approach (Kvale & Brinkmann 2015). Interviews were conducted in accessible, quiet and familiar environments selected according to participants’ preferences. Interviews were conducted either in person (in participants’ homes) or telephonically, depending on participant preference and accessibility. This flexible approach ensured that participants could engage in a comfortable and familiar environment. Interview duration varied according to participants’ communication styles, physical conditions and levels of expressiveness, ranging from 45 min – 1.5 h. The interview process evolved as new insights emerged, allowing deeper exploration of key themes (Patton 2023). With participants’ consent, interviews were audio-recorded and later transcribed verbatim to ensure accuracy (Creswell & Creswell 2017). Transcripts were anonymised by replacing identifiable information with participant codes (P1–P8). All data were stored securely on password-protected devices accessible only to the researcher.

#### Photovoice

Photovoice was employed as a participatory method to capture participants’ lived experiences visually (Vaughn & Jacquez 2020; Wang & Burris 1997). The method enables participants to document and reflect on their social realities through photographs and narrative discussion, positioning them as active contributors in the research process (Liebenberg 2018). Participants were invited to submit four photographs that represent empowerment, social inclusion, employment and well-being. Participants submitted their photographs electronically (e.g. via WhatsApp), after which the images were discussed during interviews to explore their meaning and relevance. The photographs were discussed during interviews, enabling participants to explain their meanings and contextual relevance. This method positioned participants as active contributors and enriched the dataset by capturing dimensions of experience not always accessible through verbal accounts alone.

#### Document analysis

Document analysis was used as a supplementary data source to contextualise participants’ experiences (Bowen 2009; Flick 2022). Documents analysed included training materials such as manuals, handouts and programme guidelines to provide contextual understanding of the training content and intended outcomes. Relevant training materials and programme-related documents were reviewed to gain insight into the structure, content and intended outcomes of the training interventions. Formal permission to access these documents was obtained.

#### Field notes

Field notes were used to capture observations, contextual information and analytic reflections throughout the research process (Emerson, Fretz & Shaw 2011; Flick 2022). These notes supported reflexivity, enhanced contextual understanding and contributed to analytic depth.

### Data analysis

Reflexive thematic analysis was used to analyse the qualitative data. This inductive approach involves identifying, organising and interpreting patterns of meaning across the dataset to generate themes that capture shared and divergent experiences (Braun & Clarke 2021). Audio-recorded interviews were transcribed verbatim and reviewed repeatedly to ensure familiarisation and accuracy. Transcripts were segmented into meaningful units and systematically coded. Related codes were grouped into categories and relationships between categories were explored to construct overarching themes. Themes were refined and organised to ensure coherence and alignment with the research questions (Braun & Clarke 2013; McMillan & Schumacher 2014).

### Researcher reflexivity

The researcher has professional experience in training coordination within the South African homebuilding sector. This background not only facilitated contextual understanding and rapport with participants but also required ongoing reflexivity to minimise potential bias. Reflexivity involves critically examining how the researcher’s assumptions, positionality and prior experiences may influence the research process and interpretation of findings (Flick 2022; Lichtman 2023). Reflexive field notes were maintained throughout data collection and analysis to support ongoing reflection on the researcher’s assumptions and their potential influence on the research process. Such reflexive practices enhance transparency and contribute to the credibility and trustworthiness of qualitative research.

### Measures of trustworthiness

Trustworthiness is central to evaluating the quality and rigour of qualitative research (Cypress 2017). This study adopted Lincoln and Guba’s framework, encompassing credibility, dependability, confirmability and transferability (Guba & Lincoln 1989). Credibility was enhanced through prolonged engagement, member checking and triangulation across multiple data sources, strengthening the robustness of the findings (Cope 2013; Forero et al. 2018; Guba & Lincoln 1989; Williams & Morrow 2014).

### Bias

Interview bias was minimised through the researcher’s neutral stance, avoidance of leading questions and careful management of verbal and non-verbal cues (Frey 2018). Interviews were audio-recorded and transcribed verbatim to preserve participants’ narratives (Creswell & Creswell 2017). Respondent bias was addressed by emphasising voluntary participation, confidentiality and anonymity and by conducting interviews in a language familiar to participants (Flick 2022).

### Ethical considerations

Approval was obtained from the Faculty of Education Research Ethics Committee, University of Johannesburg (Permit Number: SEM 2-2023-049). The study adhered to the ethical principles of the Declaration of Helsinki of 2013 (World Medical Association 2013). In addition, the ethical principles guiding research involving PWDs emphasise respect for dignity, autonomy, privacy and protection from harm. Informed consent was obtained voluntarily, and participants were informed of their right to withdraw at any stage. Written informed consent was obtained from all participants. Confidentiality and anonymity were strictly maintained. Participant well-being was prioritised through respectful interviewing, rapport-building, debriefing opportunities and referral to counselling services where required.

## Results

The results indicate that training contributed positively to the social and personal development of PWDs within the homebuilding industry (see Figure 3). Overall, interview narratives suggested increased confidence, autonomy and decision-making capacity, indicating a shift away from dependency. While the training environment fostered a sense of inclusion and validation, broader social participation remained constrained by persistent structural and attitudinal barriers. Accounts from participants further indicated improvements in psychological well-being and quality of life, characterised by enhanced self-worth, purpose and emotional resilience. Although immediate economic outcomes were not consistently evident, the training appeared to support longer-term aspirations related to economic participation and sustained social engagement.

## Presenting themes

Through a systematic thematic analysis of the dataset, three overarching themes were identified that reflect the social contributions of entrepreneurship training for PWDs in the South African homebuilding industry. To ensure methodological rigour and address the complexities of the data, this presentation of findings moves beyond simple description to interpretive analysis. Raw data extracts are embedded within an analytic narrative to illustrate the prevalence of the themes and demonstrate how the findings answer the core research questions. Moving beyond immediate economic metrics, these themes capture the profound personal and social transformations experienced by the participants. The three themes are: (1) Reclaiming agency in a restrictive environment; (2) beyond physical presence: The struggle against tokenism; and (3) redefining ‘results’: Psychological resilience as the true currency of training (see Table 1). Collectively, these themes illustrate how the training intersected with the participants’ psychological experiences, social positioning and structural realities.

**TABLE 1:** Synthesis of thematic findings.

Overarching theme	Sub-themes	Central concept and/or interpretative summary	Theoretical integration	Exemplar quote or raw data
1. Reclaiming agency in a restrictive environment	1.1 Internalising ‘hephapreneurship’	Participants experienced a profound psychological shift from dependency to self-sufficiency, gaining confidence and autonomy to make self-directed decisions.	**CCH Framework:** Demonstrates the capacity to lead economic empowerment. **Kirkpatrick Levels 2 and 3:** Skills translated into autonomous behaviour change.	‘Training gave me back that sense of self, knowing that I am still capable of handling responsibility in a work environment.’ (P8, female, 37 years old, Paraplegic, GP)
	1.2 The friction of an inaccessible world	The internal empowerment gained through training is frequently hindered by external structural barriers (transportation and buildings) and deep-seated societal stigmas.	**Translation Gap:** Highlights the discrepancy between individual readiness to work and systemic exclusion.	‘Even though we try to break the myth, many view someone in a wheelchair as cursed.’ (P8, female, 37 years old, Paraplegic, GP)
2. Beyond physical presence: The struggle against tokenism	2.1 The illusion of compliance	Participants felt used by the government and organisations merely to satisfy diversity quotas, resulting in performative inclusion rather than substantive integration.	**CCH Framework:** Exclusionary practices restrict navigation of the entrepreneurial landscape and perpetuate marginalisation.	Photovoice metaphors: ‘The lemon tree’ (P4, male, 38 years old, Epilepsy, NW); ‘A bucket against a wall’; ‘We as PWDs are only there for the government to tick the box of inclusion.’ (P2, male, 46 years old, Hearing Impairment, GP)
	2.2 The demand for genuine dialogue	Participants expressed frustration at being sidelined and demanded recognition as knowledgeable stakeholders who actively shape decisions and policies.	**CCH framework:** Meaningful career construction requires environments that move voices from the periphery to the centre.	‘Integration is enlightening for both disabled and non-disabled groups; we learn from each other, demonstrating our mutual interdependence.’ (P1, male, 41 years old, Hearing Impairment, GP)
3. Redefining ‘results’: Psychological resilience as the true currency of training	3.1 The ‘vitamin for the emotional Immune System’	The training successfully countered internalised stigma, restoring dignity, emotional fortitude and a renewed sense of purpose to survive an exclusionary market.	**CCH framework:** Validates that the psychological and emotional dimensions of career development are as critical as technical skills.	‘Gratitude is the vitamin for my emotional immune system. It inoculates me against the bruises of life, by reminding me I have enough. I am enough.’ (P6, female, 49 years old, Multiple Sclerosis, WC)
	3.2 Adaptability over immediate capital	Despite mixed immediate economic or financial outcomes in the homebuilding sector, participants utilised transferable skills to pivot towards other industries.	**Kirkpatrick Level 4 (Results):** Redefines long-term ‘results’ as proactive agency and long-term entrepreneurial adaptability rather than immediate profit.	‘I can use what I have to do anything.’ (P5, female, 53 years old, Paraplegic, GP)

CCH, career construction for hephapreneurship.

### Theme 1: Reclaiming agency in a restrictive environment

This overarching theme captures the central tension experienced by participants: the empowerment and newfound internal self-sufficiency gained through the training, contrasted sharply against the external, disabling realities of their environments.

#### Sub-theme 1.1: Internalising ‘hephapreneurship’

The majority of participants (P1, P2, P4, P5, P7, and P8) reported that the most significant outcome of the training was a profound psychological shift from dependency to self-sufficiency. The inclusive nature of the STEP training environment fostered a space where participants felt recognised as capable individuals rather than passive recipients of support. This internal shift strongly aligns with the CCH framework, which posits that PWDs can take charge of their economic empowerment and redefine their career trajectories. For these participants, empowerment was experienced as increased confidence, agency and control over personal decision-making. As Participant 8 articulated: ‘Training gave me back that sense of self, knowing that I am still capable of handling responsibility in a work environment’.

The acquisition of practical skills relevant to the homebuilding industry directly facilitated Kirkpatrick’s Level 2 (Learning) and Level 3 (Behaviour Change) outcomes, empowering participants to make informed decisions and manage their entrepreneurial goals autonomously. Independence, for these participants, meant a reduction in reliance on others.

One participant encapsulated this sentiment: ‘To be independent for a person with a disability is a win for yourself, not depending on other people’ (P3, female, 42 years old, Cerebral Palsy, GP). This empowerment frequently extended into their broader lives, fostering proactive engagement with the community and family. This participant also shared how the inclusion she felt translated to her family, noting that when her daughter made flowers for a funeral, ‘The sense of inclusion she felt in having this responsibility made her forget that she has a disability’ (P3, female, 42 years old, Cerebral Palsy, GP).

#### Sub-theme 1.2: The friction of an inaccessible world

Inclusive environments depend on equitable access, inclusive design and the removal of physical, technological and attitudinal barriers (Halder & Argyropoulos 2019; Syarifuddin & Al Haddar 2023). While the training successfully built internal capacity and confidence, participants consistently reported that structural and attitudinal barriers severely hindered their ability to exercise this independence. Standard TA emphasises the importance of exploring all dimensions of a phenomenon, including the challenges. In this case, the analysis revealed a pervasive ‘translation gap’: the participants’ readiness to work was frequently nullified by systemic exclusion. Despite legislative frameworks designed to protect their rights, fundamental accessibility to buildings and transport remains a significant physical barrier. South Africa’s constitutional framework guarantees equality and protection against unfair discrimination (Republic of South Africa 1996). However, participants emphasised that the most restrictive barriers were often attitudinal. Pervasive societal stigma and discriminatory attitudes continue to undermine the perceived competence of PWDs, particularly in construction-related contexts. One participant provided a powerful illustration of how deep-seated these cultural biases remain, even within their own communities: ‘Even though we try to break the myth, many view someone in a wheelchair as cursed’ (P8, female, 37 years old, Paraplegic, GP). Rather than viewing assistive devices as symbols of limitation, participants reframed them as essential tools for participation. A participant highlighted the necessity of these accommodations, explaining that an adapted car and wheelchair are ‘only modes of transportation, but to us it is essential’ (P8, female, 37 years old, Paraplegic, GP). Ultimately, this sub-theme demonstrates that while the training cultivated a strong ‘hephapreneurial’ mindset, translating this agency into sustained economic participation requires dismantling the persistent biases and physical barriers that treat PWDs as incapable.

### Theme 2: Beyond physical presence: The struggle against tokenism

This overarching theme captures the participants’ shared understanding that true social inclusion requires far more than mere physical presence or policy mandates. Through thematic analysis, it became evident that while the training fostered internal empowerment, participants consistently encountered environments in which inclusion was performative rather than substantive. For these participants, inclusion necessitates genuine recognition, access to resources and a legitimate voice in social and economic spaces.

#### Sub-theme 2.1: The illusion of compliance

The majority of participants reported that, despite existing national frameworks and policies, their inclusion in the workforce and broader society often felt like a superficial exercise. Multiple participants utilised vivid visual metaphors through the photovoice methodology to illustrate the deep frustration of being used to satisfy diversity quotas without experiencing genuine integration. One participant captured this sentiment of performative inclusion powerfully with a photograph of a bucket against a wall (see Figure 2), stating: ‘We as PWDs are only there for the government to tick the box of inclusion’ (P2, male, 46 years old, Hearing Impairment, GP). This disconnect between policy and practice leaves PWDs feeling marginalised and instrumentalised.

**FIGURE 1:**
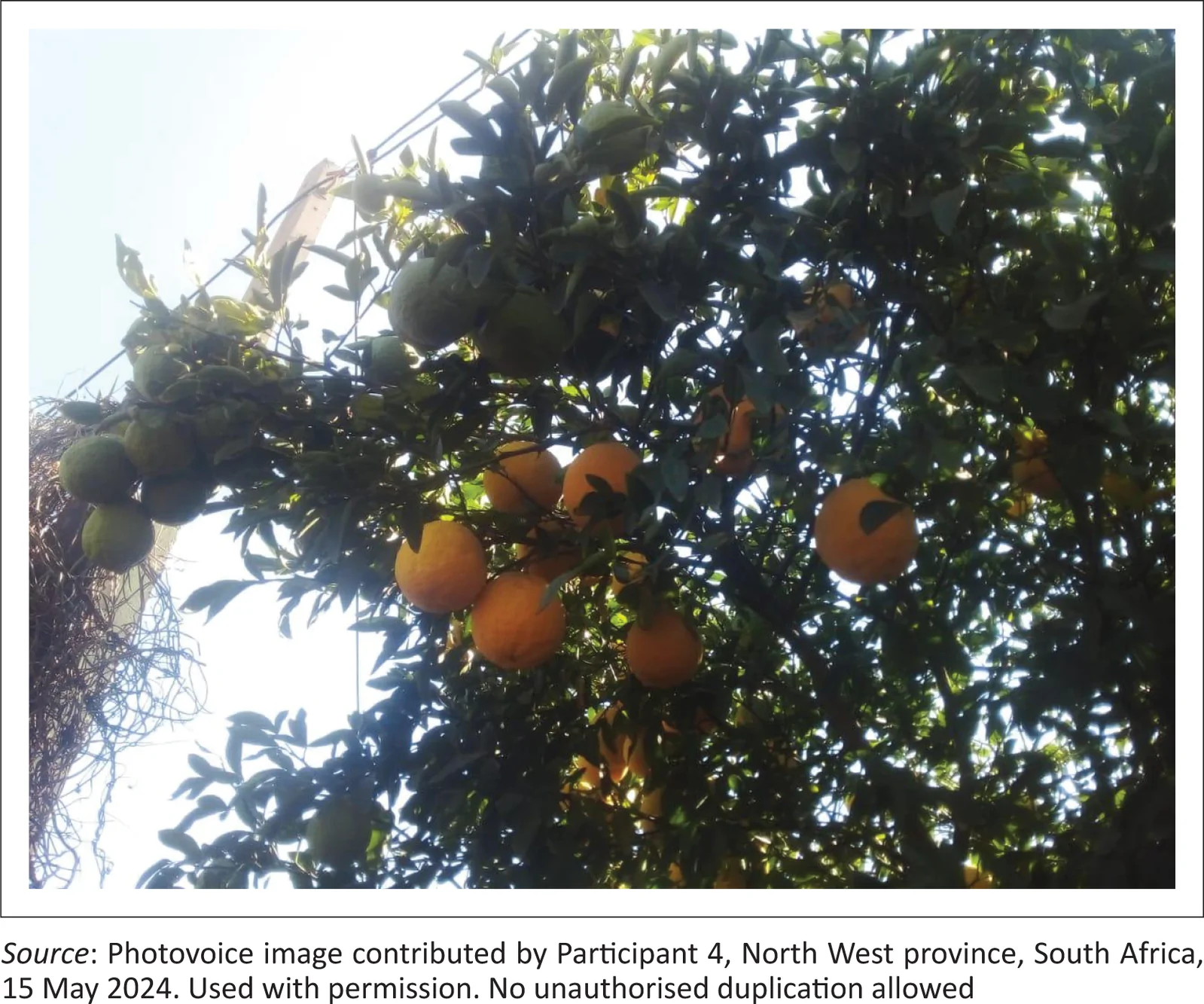
The lemon tree.

This tokenism was further illustrated by Participant 4, who submitted an image of a lemon tree bearing both green and ripe fruit. In explaining the metaphor, one participant highlighted how systemic biases position PWDs as eternally unready or incapable (see Figure 1): ‘We are just a “by the way.” We are left always green and those without disabilities are ripe, although we are all from the same community’ (P4, male, 38 years old, Epilepsy, NW).

The emotional toll of this illusion of compliance is severe. Participant 4 elaborated on this reality with another image of a water drum converted into a tool storage room, symbolising how PWDs are locked away and only brought out when organisations need to prove regulatory compliance:

‘They will only come to me when they need to use me, and after that they can throw me anywhere till I start tripping them and irritating them again, then they will take me and lock me in the drum… People always come to me to use me for their own legal compliance and thereafter dump me or lock me a drum and continue with their lives as if I don’t exist.’ (P8, female, 37 years old, Paraplegic, GP)

Viewed through the CCH framework, these exclusionary practices actively restrict the participants’ ability to navigate the entrepreneurial landscape and assert their economic independence. The illusion of compliance perpetuates stigma and isolates PWDs, limiting their potential to contribute meaningfully to society.

#### Sub-theme 2.2: The demand for genuine dialogue

In direct contrast to the isolation of tokenism, this sub-theme captures the participants’ strong desire for authentic integration and shared decision-making. Participants expressed significant frustration over being unheard and sidelined in matters that directly affect their lives and well-being. The analysis revealed that true social inclusion requires recognising PWDs as knowledgeable stakeholders who actively shape dialogue. Instead of being treated as active contributors, participants felt their input was frequently ignored or tokenised, reinforcing barriers to equity. However, participants also articulated a clear, positive vision for what genuine dialogue could achieve. One participant emphasised the mutual benefits of authentic integration, noting, ‘Integration is enlightening for both disabled and non-disabled groups; we learn from each other, demonstrating our mutual interdependence’ (P1, male, 41 years old, Hearing Impairment, GP).

**FIGURE 2:**
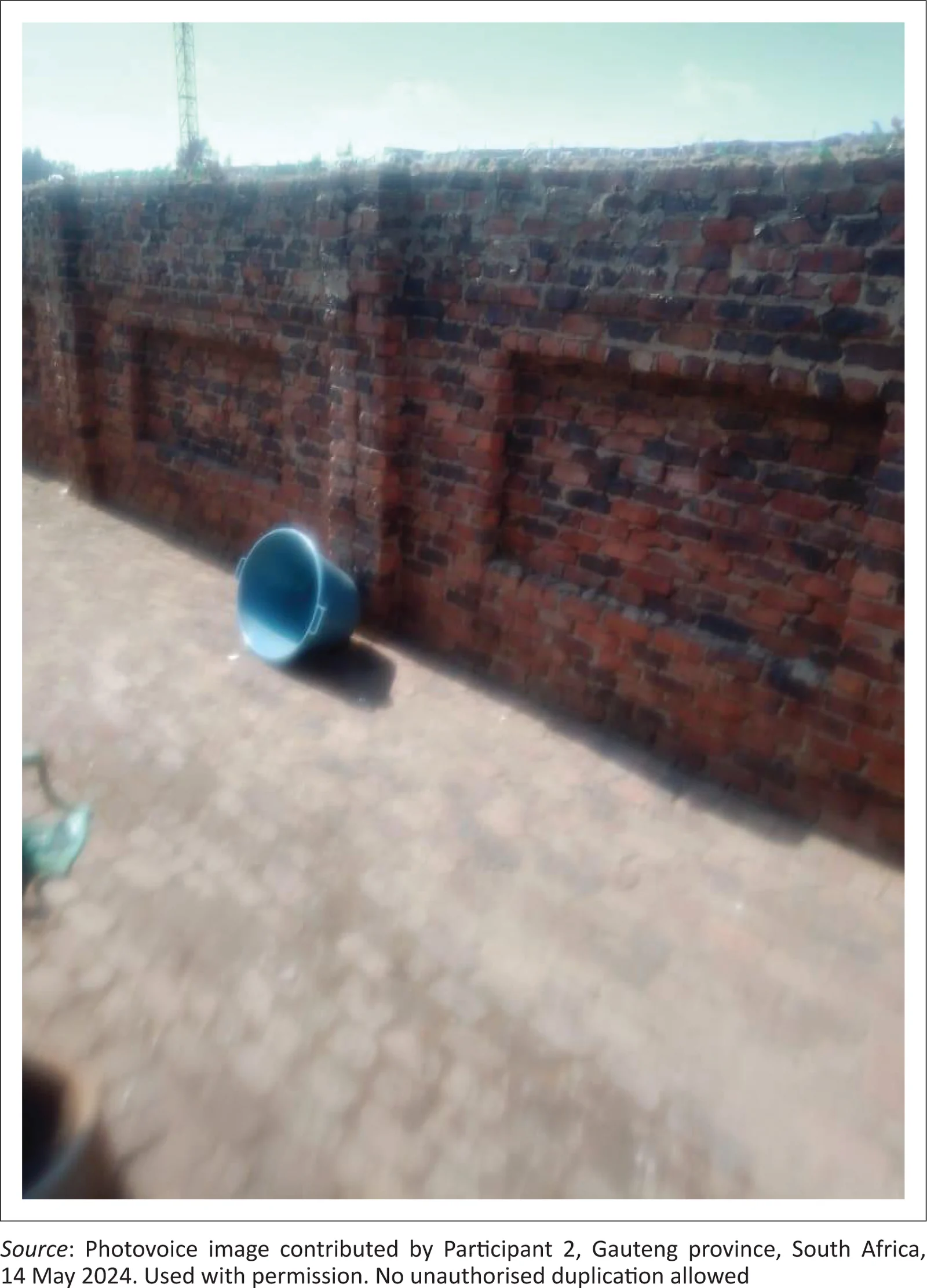
Bucket against a wall.

**FIGURE 3:**
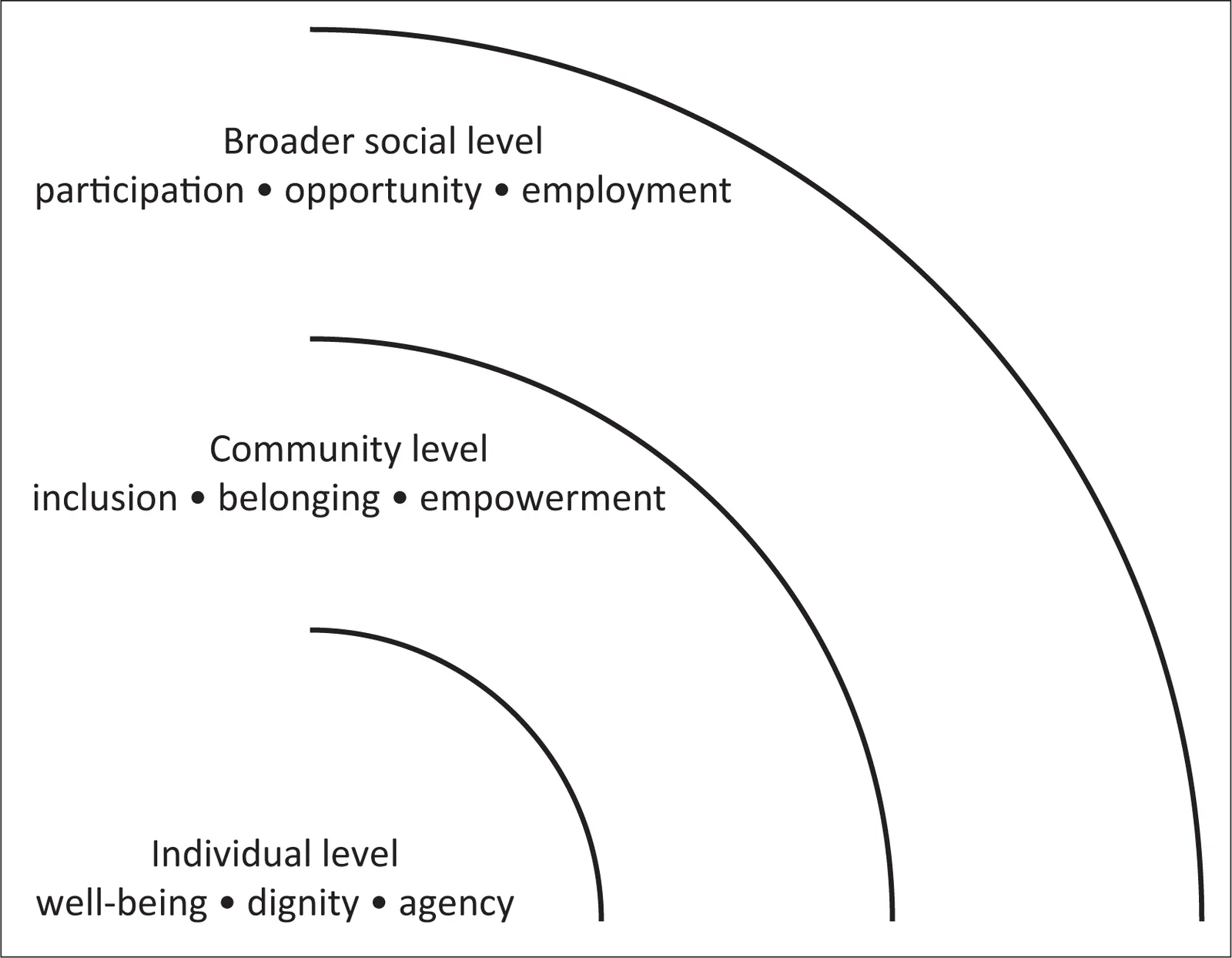
Multilevel social contributions of training for persons with disabilities.

Participants advocated for platforms where their collective voices could drive systemic change. One participant shared a photovoice image of chocolate bunnies titled ‘The gossip’ to represent the power of community among PWDs, stating: ‘You can be different in a good way to make a big difference as a group’ (P7, male, 35 years old, Traumatic Brain Injury, WC).

Ultimately, this sub-theme demonstrates that social inclusion cannot be achieved through passive presence alone. As aligned with the CCH framework, participants require inclusive environments and robust advocacy to overcome societal stigma, ensuring their voices move from the periphery to the centre of policy and organisational planning.

### Theme 3: Redefining ‘results’: Psychological resilience as the true currency of training

The final overarching theme captures the ultimate, long-term outcomes of the entrepreneurship training. Standard thematic analysis requires looking beyond surface-level assumptions to understand the complex realities of the participants. While the original intention of the training was largely focused on business creation, the analysis revealed that participants redefined what constituted a successful ‘result’. Rather than immediate financial capital, the true currency gained from the training was psychological resilience, renewed purpose and the adaptability to survive an exclusionary labour market.

#### Sub-theme 3.1: The ‘vitamin for the emotional immune system’

The majority of participants reported that the most profound impact of the training was a significant improvement in their psychological well-being and overall quality of life. Before the intervention, many participants described facing intense societal marginalisation that constrained their self-worth. However, engagement in the training successfully countered this internalised stigma and reinforced a strong belief in their inherent capabilities.

This psychological empowerment is best encapsulated by one participant, who highlighted the emotional fortitude gained from the experience: ‘Gratitude is the vitamin for my emotional immune system. It inoculates me against the bruises of life, by reminding me I have enough. I am enough’ (P6, female, 49 years old, Multiple Sclerosis, WC). Similarly, the training acted as a catalyst for breaking cycles of isolation. As one participant observed: ‘When stagnancy is disrupted in someone’s life, it opens a whole new world and whole new possibilities’ (P8, female, 37 years old, Paraplegic, GP), while another participant noted: ‘Before the training, I felt stuck. Now, mentally, I feel stronger and more positive about my future’ (P5, female, 53 years old, Paraplegic, GP).

These findings align strongly with the CCH framework, which recognises that the emotional dimensions of career development are just as critical as technical skills. The framework posits that personal fulfilment and psychological empowerment are key outcomes of career construction. For these participants, the training provided the emotional resilience required to sustain long-term engagement in community and economic participation.

#### Sub-theme 3.2: Adaptability over immediate capital

A rigorous thematic analysis must also account for mixed or divergent data. When analysing the direct economic opportunities generated by the training, the outcomes were highly mixed. Some participants, such as Participant 1 and Participant 4, expressed frustration regarding the lack of immediate, tangible financial benefits and highlighted a critical need for structured support in securing funding or tenders within the homebuilding sector. However, rather than viewing this lack of immediate capital as a failure, the analysis demonstrates how participants utilised the competencies acquired to pivot towards other industries. Because the training focused heavily on transferable skills, participants developed a proactive adaptability. One participant illustrated this expanded mindset, stating: ‘I can use what I have to do anything’ (P5, female, 53 years old, Paraplegic, GP). For others, like Participant 6, immediate financial gain was never the primary motivation, as she prioritised the personal growth and fulfilment derived from entrepreneurial activities. Evaluated through Level 4 (Results) of Kirkpatrick’s model of training evaluation, which assesses the overall, long-term impact on the trainees, this sub-theme demonstrates a highly successful outcome. While the direct economic opportunities in the homebuilding sector were limited, the true ‘result’ was the behavioural shift towards proactive agency. By leveraging their transferable skills for broader applications, the participants secured the long-term adaptability necessary for future entrepreneurial survival.

## Discussion

The findings of this study demonstrate that entrepreneurship training within the South African homebuilding industry functions as a multifaceted catalyst for personal and social transformation among PWDs. Through a systematic thematic analysis, this research reveals that while the training successfully builds individual capacity and psychological resilience, a pervasive ‘translation gap’ remains. The ability of PWDs to apply their newfound skills in the broader economy is often undermined by systemic, structural and attitudinal barriers. These findings hold significant implications for how disability entrepreneurship is conceptualised, evaluated and supported.

### Reclaiming agency and the ‘translation gap’

The theme of *reclaiming agency in a restrictive environment* highlights empowerment as a relational, multidimensional process wherein PWDs transition from dependency to self-directed decision-making. The findings confirm that inclusive training environments, which normalise disability and provide tailored accommodations, are essential for fostering this internal shift. This aligns with assertions by Carter et al. (2023), who note that inclusive programme design directly enhances participation and long-term outcomes for marginalised groups. Evaluated through the CCH framework, this psychological shift exemplifies ‘hephapreneurship’ or the capacity of PWDs to take charge of their economic destinies. However, the study also foregrounds a critical tension: internal empowerment is insufficient without enabling external environments. Participants consistently reported that inaccessible transport, rigid infrastructure and entrenched societal stigmas severely restricted their ability to translate Kirkpatrick’s Level 2 (Learning) and Level 3 (Behaviour Change) into sustained economic practice. These findings echo broader literature emphasising that physical and cultural barriers compound to limit PWDs professional growth (Enginöz & Şavlı 2016). Consequently, future empowerment initiatives must extend beyond individual skill acquisition to aggressively tackle the environmental and social barriers that enforce this ‘translation gap’.

### Tokenism and the demand for genuine dialogue

The findings captured under *beyond physical presence: the struggle against tokenism* reveal that true social inclusion requires far more than physical integration or symbolic compliance. While national policy frameworks, such as the White Paper on the Rights of Persons with Disabilities, advocate for inclusion, participants experienced a profound disconnect between policy intent and implementation (Department of Social Development 2016). Participant metaphors, such as being treated as the unharvested ‘green fruit’ or a ‘bucket against a wall’, vividly illustrate the frustration of performative inclusion. These experiences support Dineshan’s and Geetha (2025) critique regarding the weak enforcement of disability inclusion frameworks, which often results in PWDs being used merely to satisfy organisational diversity quotas rather than being valued as substantive contributors. As Thomas et al. (2020) argue, sustainable inclusion is impossible without genuine representation and shared decision-making. For social inclusion to transition from an illusion to a reality, policies must recognise PWDs as knowledgeable stakeholders who actively shape the dialogue and practices that impact their lives and entrepreneurial ventures.

### Redefining ‘results’ through psychological resilience

When evaluating the training outcomes at Level 4 (Results) of Kirkpatrick’s model, the findings necessitate a redefinition of entrepreneurial success. While traditional vocational assessments prioritise immediate financial capital or direct employment, this study found that the most profound ‘results’ were non-economic: enhanced emotional fortitude, renewed purpose and long-term adaptability. For many participants, the training acted as a ‘*vitamin for the emotional immune system*’, equipping them with the psychological resilience necessary to navigate an exclusionary labour market. This aligns with the findings of Kotze and Massyn (2019), who argue that holistic approaches to well-being – incorporating social and emotional dimensions – are critical for fostering resilience and fulfilment. Furthermore, despite mixed immediate financial outcomes in the homebuilding sector, the transferable skills acquired enabled participants to pivot proactively to alternative industries. These findings suggest that entrepreneurship programmes for PWDs must be evaluated through a broader, more holistic lens. As the CCH framework posits, the emotional dimensions of career development are as critical as technical skills. Measuring success purely in economic terms obscures the profound psychological and social impacts that underpin future economic participation and community integration.

### Implications of the study

This study highlights the significance of training as a legitimate pathway for empowerment, social inclusion and economic participation among PWDs. The results challenge persistent assumptions that participation in skills-based and vocational training – particularly within sectors such as homebuilding – is unrealistic or inappropriate for PWDs. Instead, the results demonstrate PWDs capacity to contribute meaningfully to economic and community development when enabling conditions are in place. Training is thus reframed not merely as a mechanism for skill acquisition, but as a social process through which dignity, agency and participation can be realised. The results further suggest that training interventions hold implications beyond technical competence. Empowerment emerged as a relational and contextual process shaped by confidence, autonomy and the affirmation of capability. This highlights the importance of understanding training environments as social spaces that can either enable or constrain self-determination. Training, therefore, has broader implications for how inclusion is conceptualised, extending beyond access to skills towards recognition, participation and voice. Persistent structural barriers (including inaccessible infrastructure, limited resources and entrenched societal attitudes) remain central factors influencing the translation of training into meaningful outcomes. The presence of these barriers highlights the gap between policy intent and lived experience and illustrates how inclusion may remain symbolic when not supported by enabling environments. These results contribute to broader debates on disability inclusion by demonstrating that economic participation is shaped by structural and attitudinal contexts rather than individual capability alone. Finally, the study emphasises the importance of viewing training as part of a broader inclusion ecosystem. The social contributions identified (such as increased confidence, improved well-being and enhanced social participation) suggest that training interventions have implications for inclusive economic development strategies that recognise PWDs as active contributors rather than passive beneficiaries. These insights extend current scholarship by foregrounding the social and human development dimensions of training within disability contexts.

### Strengths and limitations

A key methodological strength of this study was the use of photovoice, a participatory method that empowered participants as active co-researchers to capture dimensions of exclusion often missed in traditional interviews visually. The qualitative multiple-case design provided rich, contextually grounded insights into the lived realities of PWDs across diverse provinces and real-world settings. However, the study has several limitations. The focus on only three South African provinces limit the generalisability of the results. Experiences in other regions may differ because of variations in local governance capacity and resource availability. In addition, because the results rely on self-reported narratives, they may be influenced by personal interpretation or social desirability bias.

### Recommendations

The results highlight important implications for the design, implementation and evaluation of training initiatives for PWDs. While such training demonstrates clear potential to enhance empowerment, social inclusion and well-being, its impact remains constrained when systemic barriers persist. This emphasises the importance of situating training within broader inclusive ecosystems that address accessibility, policy implementation and sustained post-training support. A key implication is the continued lack of accessible infrastructure and transport, which restricts participation in training, employment and economic activity. Without physically accessible training venues, workspaces and marketplaces, gains in individual capability remain vulnerable to environmental exclusion. The results further point to challenges in policy implementation. Although disability inclusion frameworks exist, inconsistent enforcement and limited awareness undermine their practical impact. This stresses the need for stronger accountability mechanisms and the meaningful inclusion of PWD voices to move beyond symbolic compliance towards genuine participation. Training programmes also carry important implications for inclusive design. Flexible delivery approaches, accessible learning materials and appropriately trained facilitators are essential, alongside mentorship, networking and peer-support mechanisms to strengthen sustainability and social inclusion. Access to finance remains a critical structural factor, with tailored funding mechanisms and financial literacy support shaping longer-term economic participation. Finally, the study highlights the value of ongoing monitoring and evaluation. Longitudinal assessment of empowerment, economic participation and well-being can strengthen evidence of programme impact and inform continuous improvement. Future research may further explore digital training modalities, intersectionality and comparative implementation models to advance inclusive economic participation.

## Conclusion

This study examined the social contributions of training for PWDs within South Africa’s homebuilding industry, focusing on empowerment, social inclusion, economic participation and overall well-being. The results demonstrate that training can function as a meaningful catalyst for personal and social transformation, particularly by enhancing confidence, self-efficacy and independent decision-making. Participants reported increased agency and a shift away from dependency, highlighting the role of inclusive training environments in supporting empowerment and capability development. Social inclusion emerged as a significant outcome, with training facilitating a greater sense of belonging and participation. While the programme enabled participants to challenge stereotypes and engage more actively within their communities, persistent barriers – such as inaccessible infrastructure, limited resources and societal stigma – continued to constrain full inclusion. These challenges underline the need for enabling environments that extend beyond training contexts. Although immediate employment and financial outcomes were not consistently evident, the training strengthened participants’ adaptability and resilience, enabling them to explore diverse economic and vocational pathways. Importantly, the study also identified positive effects on psychological well-being and quality of life, including enhanced self-worth, purpose and emotional resilience. Overall, the results affirm that training holds significant potential to advance the empowerment and social participation of PWDs. However, its impact is maximised when combined with accessible infrastructure, effective policy implementation and inclusive societal attitudes. This study contributes to disability scholarship by foregrounding the social and human development outcomes of training and provides a foundation for future research and practice aimed at fostering inclusive and sustainable participation in economic and community life.
